# Data on how several physiological parameters of stored red blood cells are similar in glucose 6-phosphate dehydrogenase deficient and sufficient donors

**DOI:** 10.1016/j.dib.2016.06.018

**Published:** 2016-06-23

**Authors:** Vassilis L. Tzounakas, Anastasios G. Kriebardis, Hara T. Georgatzakou, Leontini E. Foudoulaki-Paparizos, Monika Dzieciatkowska, Matthew J. Wither, Travis Nemkov, Kirk C. Hansen, Issidora S. Papassideri, Angelo D׳Alessandro, Marianna H. Antonelou

**Affiliations:** a Department of Biology, Section of Cell Biology and Biophysics, School of Science, NKUA, Athens 15784, Greece; b Laboratory of Hematology and Transfusion Medicine, Department of Medical Laboratories, Faculty of Health and Caring Professions, Technological and Educational Institute of Athens, Athens 12210, Greece; c Regional Blood Transfusion Center, “Agios Panteleimon” General Hospital of Nikea, Piraeus 18454, Greece; d Department of Biochemistry and Molecular Genetics, University of Colorado, School of Medicine–Anschutz Medical Campus, Aurora, 80045 CO, USA

**Keywords:** AnnV, annexin V, CPD, citrate-phosphate-dextrose, FRAP, ferric reducing antioxidant power, FSC, forward scatter, G6PD, glucose-6-phosphate dehydrogenase, G6PD^−^, G6PD deficiency, Hb, hemoglobin, Hct, hematocrit, K^+^, potassium, MCF, mean corpuscular fragility, MFI, mechanical fragility index, MP, micoparticles, microvesicles, MPPA, microparticles pro-coagulant activity, NAC, N-acetylcysteine, NS, non-stored, PBS, phosphate buffer saline, PCI, protein carbonylation index, PS, phosphatidylserine, RBC, red blood cell, RFU, relative fluorescence units, ROS, reactive oxygen species, SAGM, saline-adenine-glucose-mannitol, SSC, side scatter, TAC, total antioxidant capacity, tBHP, *tert*-Butyl hydroperoxide, UA-dep AC, uric acid dependent antioxidant capacity, UA-ind AC, uric acid independent antioxidant capacity, G6PD deficiency, Red blood cell storage lesion, Oxidative stress, Cell fragility, Microparticles

## Abstract

This article contains data on the variation in several physiological parameters of red blood cells (RBCs) donated by eligible glucose-6-phosphate dehydrogenase (G6PD) deficient donors during storage in standard blood bank conditions compared to control, G6PD sufficient (G6PD^+^) cells. Intracellular reactive oxygen species (ROS) generation, cell fragility and membrane exovesiculation were measured in RBCs throughout the storage period, with or without stimulation by oxidants, supplementation of N-acetylcysteine and energy depletion, following incubation of stored cells for 24 h at 37 °C. Apart from cell characteristics, the total or uric acid-dependent antioxidant capacity of the supernatant in addition to extracellular potassium concentration was determined in RBC units. Finally, procoagulant activity and protein carbonylation levels were measured in the microparticles population. Further information can be found in “Glucose 6-phosphate dehydrogenase deficient subjects may be better “storers” than donors of red blood cells” [1].

**Specifications Table**

**Table:** 

Subject area	Biology
More specific subject area	Biology of erythrocytes stored in blood banks for transfusion purposes
Type of data	Graphs, figures
How data was acquired	Cell fragility tests, hemolysis and total antioxidant capacity were measured spectrophotometrically. Reactive oxygen species were quantified by fluorometry. Supernatant potassium and microparticles were assayed using Elecsys Systems Analyzer (Roche) and flow cytometry, respectively. Microparticles’ pro-coagulant activity and protein carbonylation were measured by Elisa assays. Metabolomics analysis was performed by ultimate high pressure liquid chromatography-mass spectrometry coupled online with a Q Exactive system.
Data format	Analyzed
Experimental factors	Intracellular ROS generation was measured in energy depleted stored RBCs (incubation for 24 h at 37 °C). Osmotic and mechanical fragility indexes were estimated in situ or after incubation of stored RBCs at the same conditions (24 h/37 °C). Apart from microparticles’ and metabolomics analysis, all other assays were performed on day 42 samples with or without supplementation of the units with 2.5 mM N-acetylcysteine (NAC).
Experimental features	Physiological characteristics of stored RBCs and supernatants and malate variation were examined in RBC units donated by G6PD^−^ and G6PD^+^ eligible donors. Most measurements were performed at week intervals of the storage period. NAC supplementation was applied to aliquots of the RBC units on day 21 of storage and the effects were analyzed on day 42 samples.
Data source location	National and Kapodistrian University of Athens (NKUA), School of Science*,* Athens 15784, Greece
	Technological and Educational Institute of Athens, Athens 12210, Greece
	University of Colorado, School of Medicine–Anschutz Medical Campus, Aurora, 80045 CO, USA
Data accessibility	Data with this article

**Value of the data**

• The different storage profile when additional oxidative stimuli are added, is of value for the understanding of G6PD deficient red blood cells’ physiology and its clinical relevance to the transfusion׳s outcomes.
• Cell fragilities profiles and microparticles’ characteristics might help elucidating the storage capacity (“storability”) of G6PD deficient red blood cells.
• Our data contribute to the clarification of donor-variation and N-acetylcysteine supplementation effects on red blood cell storage lesion

## 1 Data

**Fig. 1:**
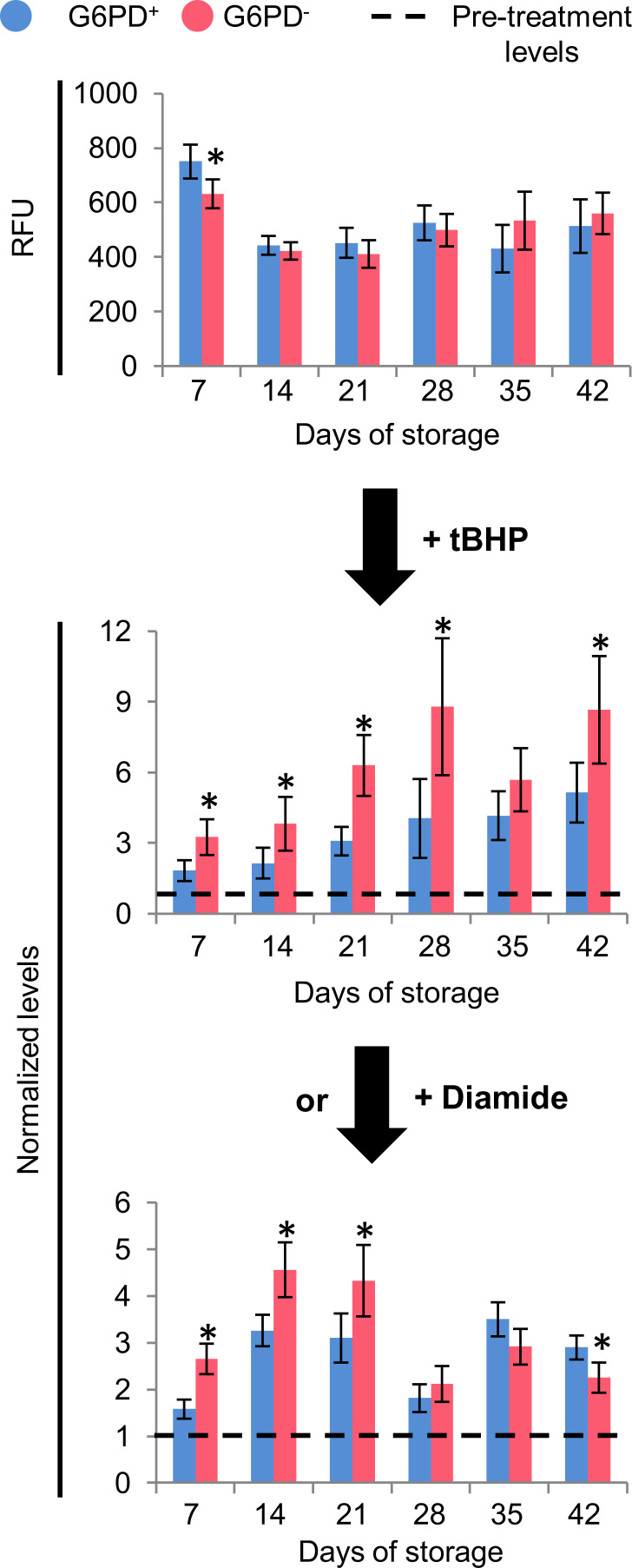
ROS generation in energy depleted G6PD-deficient (G6PD^−^ n=6) and control (G6PD^+^*n*=3) RBCs. RBCs stored for variable periods of time in CPD-SAGM preservative/additive solution were incubated for 24 h at 37 °C and intracellular ROS accumulation was estimated by fluorometry with or without stimulation by oxidants (100 μM tBHP for 20 min at 20 °C and 2 mM diamide for 45 min at 37 °C). Stimulated ROS were normalized to the pre-treatment levels (dashed lines). RFU, relative fluorescence units. *P<0.05 *versus* control (G6PD^+^) donors; data shown as mean±standard deviation.

**Fig. 2:**
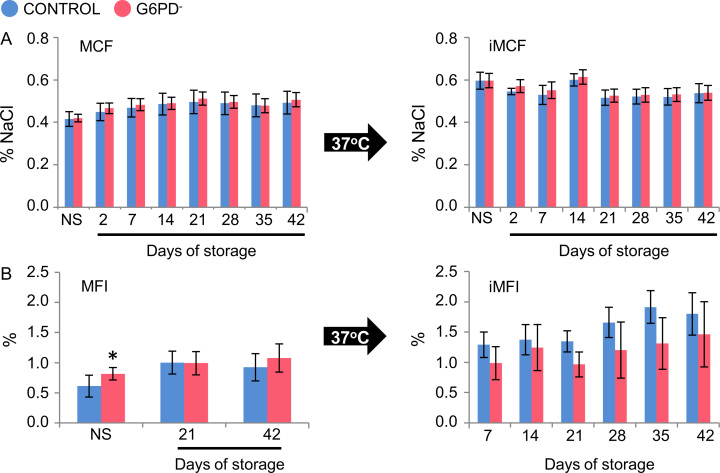
RBC fragilities profiles. Variation in mean corpuscular fragility (MCF) (A) and mechanical fragility index (MFI) (B) of G6PD^−^ (*n*=6) and control (G6PD^+^) (*n*=3) RBCs before (NS, non-stored) and during storage in CPD-SAGM. Left panels: MCF and MFI measurements *in situ* (no treatment of RBCs). Right panels: MCF and MFI measurements following incubation of non-stored and stored RBCs for 24 h at 37 °C (iMCF, iMFI). *P<0.05 *versus* control; data shown as mean±standard deviation.

**Fig. 3:**
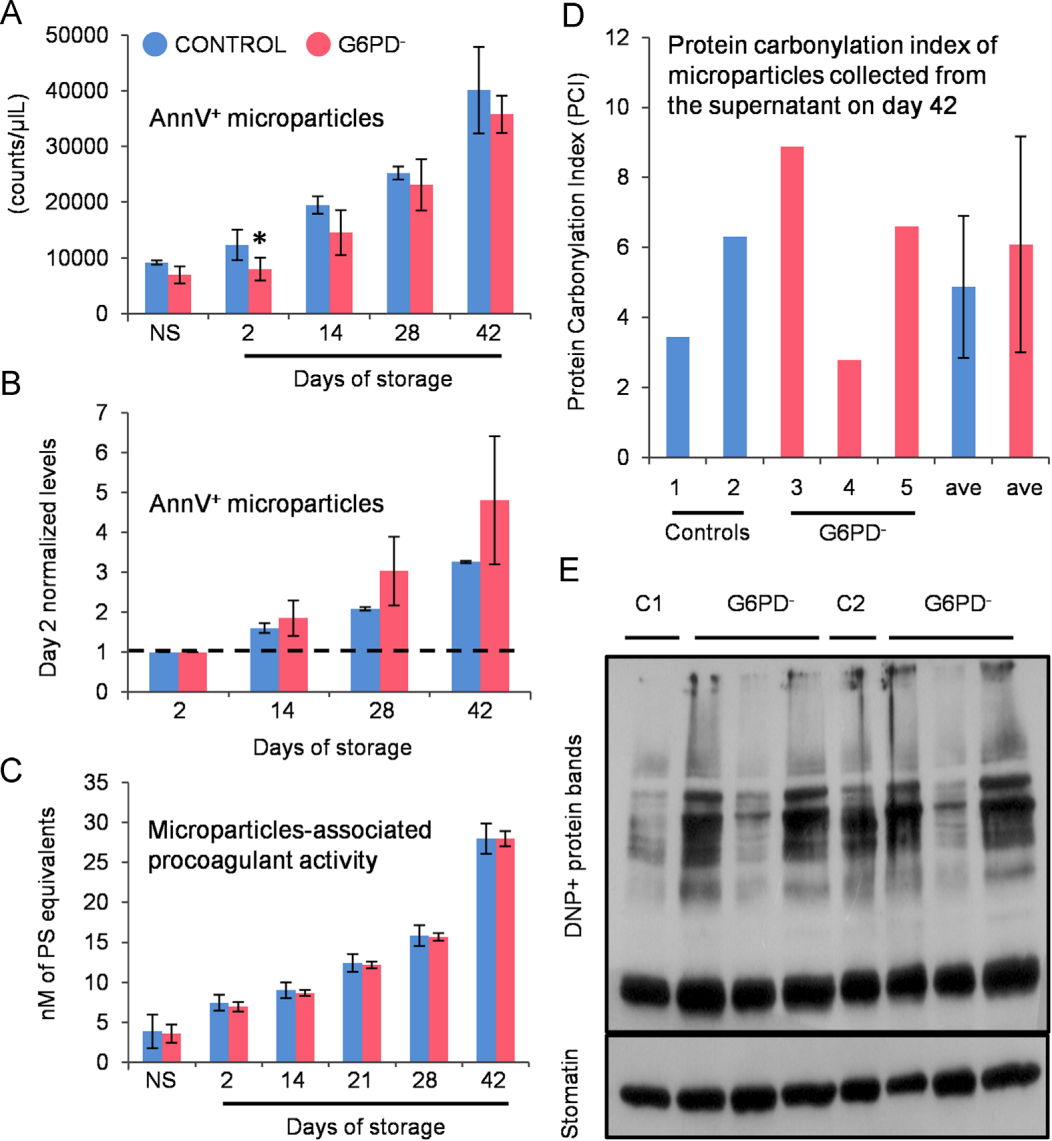
Microparticles characterization. Left panel: (A) Flow cytometry estimation of RBC-derived Annexin V-positive microparticles in control (*n*=3) and G6PD^−^ (*n*=6) samples before (non-stored, NS) and during the storage. (B) After normalization to the second day of storage (dashed line) the G6PD^−^ RBCs exhibited a trend for higher microvesiculation rate compared to the control RBCs. (C) Microparticles-associated procoagulant activity measured by Elisa was similar in the two groups under examination. *P<0.05 *versus* control; data shown as mean±standard deviation. Right panel: (D) relative percentage of carbonylated proteins in microparticles collected from G6PD^+^ (individuals # 1 and 2) and G6PD^−^ (individuals # 3–5) supernatant on the day 42 of storage and (E) representative immunoblot analysis by using anti-DNP antibody. Stomatin was used as internal control.

**Fig. 4:**
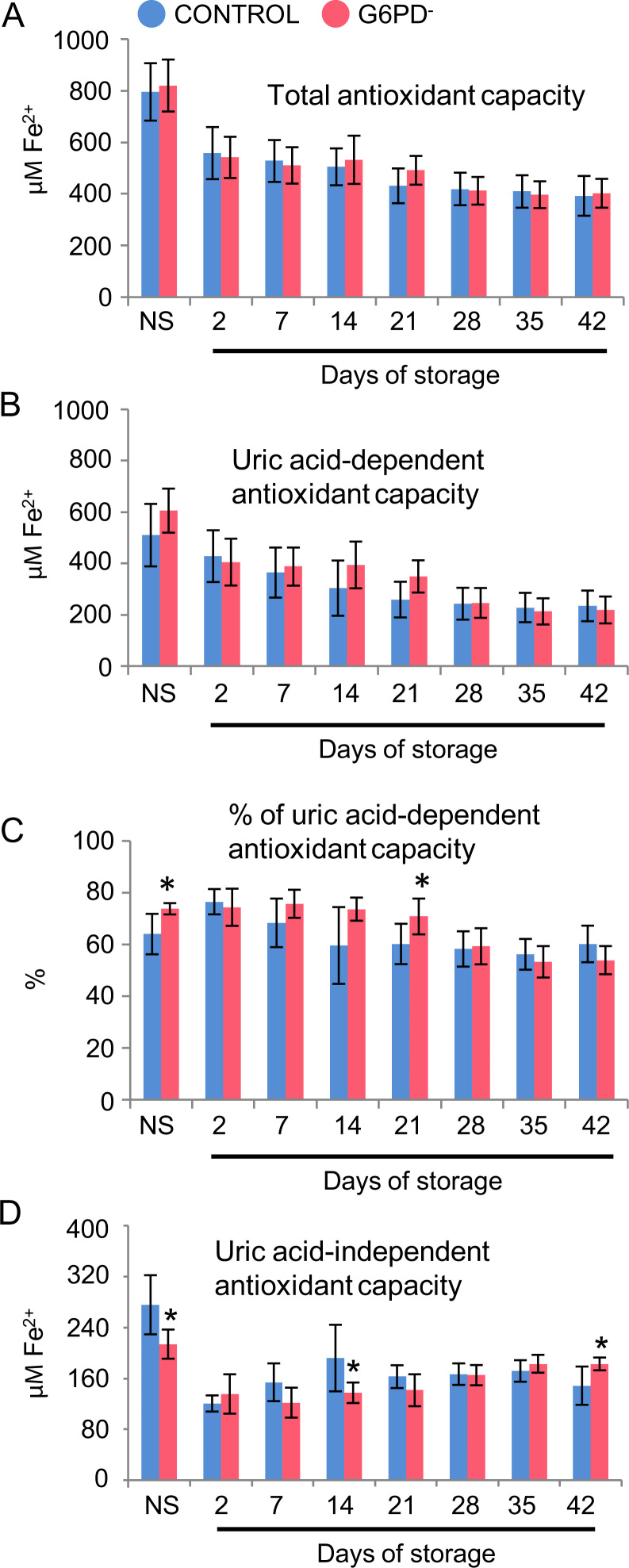
Estimation of the antioxidant capacity in fresh plasma and supernatant collected from the stored RBC units. Total, uric acid-dependent and uric acid-independent antioxidant capacity in G6PD^−^ (*n*=6) and control (G6PD^+^) (*n*=3) samples *in vivo* (non-stored, NS) and during storage. *P<0.05 *versus* control. Data is shown as mean±standard deviation.

We assessed the storage quality of red blood cells (RBCs) donated by glucose-6-phosphate dehydrogenase (G6PD) deficient, yet eligible, donors compared to control (G6PD sufficient) red blood cells [1]. Intracellular reactive oxygen species (ROS) accumulation was similar in energy depleted (24 h/37 °C) G6PD^−^ and G6PD^+^ stored RBCs while stimulation by *tert-*Butyl hydroperoxide (tBHP) and diamide oxidants resulted in statistically significant increase in ROS accumulation in the G6PD^−^ group (*n*=6) compared to the G6PD^+^ group (*n*=3) (Fig. 1). RBC fragility (both mean corpuscular fragility, MCF and mechanical fragility index, MFI) (Fig. 2) and the characteristics of the microparticles (accumulation, pro-coagulant activity and protein carbonylation index, PCI) (Fig. 3) were equal between the groups under examination throughout the storage period, while only slight differences were observed in the antioxidant capacity of the supernatant (Fig. 4).

**Fig. 5:**
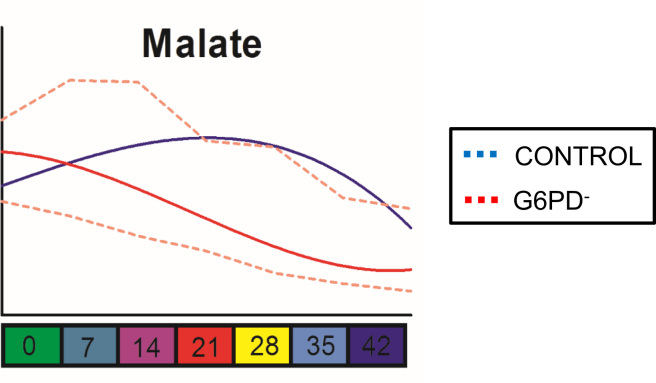
Malate variation in G6PD^−^ (*n*=6) and control (G6PD^+^, *n*=3) RBCs during storage in CPD-SAGM. Metabolomics analysis showed a faster decrease of malate levels in G6PD^−^ RBCs than in controls. Blue line: control; solid and dashed red lines: median+SD for G6PD^−^ cells.

**Fig. 6:**
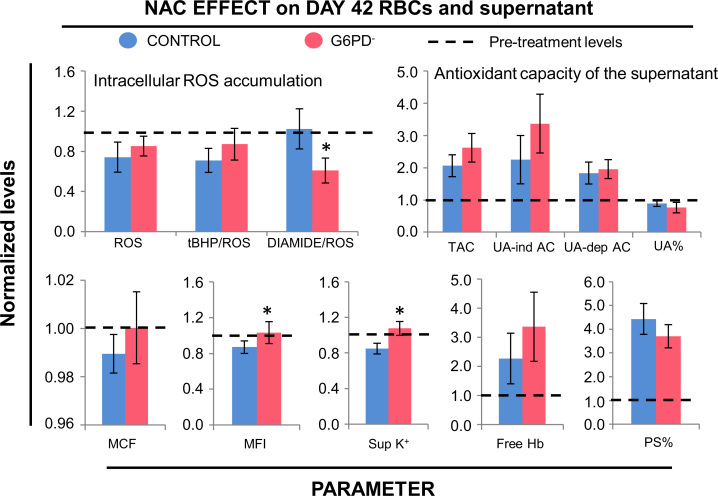
NAC effect on physiological characteristics of stored G6PD^−^ (n=6) and control (G6PD^+^, *n*=3) RBCs. Packed RBC units were treated with 2.5 mM NAC (from day 21 to day 42) and samples were collected on the last day of storage. All data are normalized to pre-treatment levels (dashed lines). *P<0.05 *versus* control. TAC, total antioxidant capacity; UA-ind AC, uric acid independent antioxidant capacity; UA-dep AC, uric acid dependent antioxidant capacity, Sup K^+^, supernatant potassium; PS, phosphatidylserine exposure at RBC surface; data shown as mean±standard deviation.

Malate levels decreased faster in G6PD^−^ RBCs than in control, G6PD^+^ RBCs during the storage period in CPD-SAGM (Fig. 5). Finally, N-acetylcysteine (NAC) supplementation (at the concentration used) induced similar changes in both stored RBCs and supernatant (Fig. 6).

## 2 Experimental design, materials and methods

### 2.1 Blood collection and processing

Blood samples from 9 regular male donors 22–30 years old (*n*=6 for Mediterranean variant grade II WHO G6PD^−^ donors and *n*=3 for donors with normal levels of G6PD activity) were collected into EDTA or citrate vacutainers. The quality of RBC concentrates prepared from the same donors was evaluated in pre-storage leukoreduced (RC High efficiency leukocyte removal filters, Haemonetics, MA, USA), citrate-phosphate-dextrose (CPD)/saline-adenine-glucose-mannitol (SAGM) units throughout a 42 days storage in a controlled environment at 4 °C as previously described [1]. Samples were taken after 2 days of storage and weekly thereafter or on days 21 and 42 (middle and maximal storage duration, respectively).

### 2.2 Redox status estimation

ROS accumulation in stored RBCs was detected with the membrane-permeable, non-fluorescent and redox-sensitive probe 5-(and-6)-chloromethyl-2′,7′-dichloro-dihydro-fluorescein diacetate, acetyl ester (CM-H_2_DCFDA) according to the manufacturer׳s guidelines (Invitrogen, Molecular Probes, Eugene, OR) with minor modifications as previously described [2]. After resuspension of RBCs at 1% hematocrit, 10 µM of CM-H_2_DCFDA were added and fluorescence was measured at 490 nm excitation and 520 nm emission wavelengths by using the VersaFluor Fluorometer System (Bio-Rad, Hercules, CA). ROS production using exogenous stimuli was calculated after incubation of RBCs with 100 µM *tert*-Butyl hydroperoxide (tBHP) for 20 min at 20 °C or 2 mM diamide for 45 min at 37 °C.

The ferric reducing antioxidant power (FRAP) assay was used for the estimation of Total Antioxidant Capacity (TAC) of the plasma [3]. Briefly, small aliquots of plasma were mixed with freshly prepared working FRAP solution (containing acetate buffer (pH 3.6, 300 mM), TPTZ (2,4,6-tripyridyl-*s*-triazine, 10 mM) in HCl (40 mM) and FeCl_3_ (20 mM) in 10:1:1 ratio) and incubated for 4 min at 37 °C in a water bath. Absorbance was measured at 593 nm. Uric acid-dependent and -independent antioxidant capacity were determined after uricase treatment of the samples (for 20 min at 25 °C) [4]. Redox status estimation tests were also performed after N-acetylcysteine (NAC) supplementation (2.5 mM) or incubation for 24 h at 37 °C.

### 2.3 Hemolysis and cellular fragility assays

Plasma free hemoglobin was calculated following a method first described by Harboe [5]. Blood samples were centrifuged at 1000×*g* for 10 min. Plasma was collected and centrifuged again under the same conditions. Cell free supernatants were diluted in distilled water and incubated at 20 °C for 30 min. Hb absorbance was measured versus blank at 380, 415 and 450 nm. The final OD was calculated as follows: 2×OD415−OD380−OD450.

*In vitro* osmotic fragility behavior of erythrocytes was determined in solutions of increasingly saline concentration (0.0–0.9% w/v NaCl) [6]. 10 µl of blood samples were added in 1.0 ml of each saline solution, incubated for 15 min at 20 °C and then centrifuged at 1500 rpm for 5 min. Hb released in the supernatant was measured at 540 nm, plotted against saline concentration and the mean corpuscular fragility (MCF), which corresponds to the saline concentration causing 50% of hemolysis, was calculated.

Mechanical fragility of RBCs was evaluated as previously described [7]. Briefly, blood from each donor was mixed with stainless steel beads, rocked in a rocker platform for 1 h and free Hb was measured in the plasma by using both Harboe׳s method against an un-rocked control. The mechanical fragility index (MFI) was calculated using the formula:

$$\mathrm{MFI}=\left[\left({\mathrm{Hb}}_{\mathrm{rocked}}–{\mathrm{Hb}}_{\mathrm{control}}\right)/\left({\mathrm{Hb}}_{\mathrm{aliquot}}–{\mathrm{Hb}}_{\mathrm{control}}\right)\right]\times 100$$

where Hb_rocked_ is the mean free Hb concentration in the supernatants of the rocked specimens, Hb_control_ is the average free Hb concentration in the supernatants of the control samples, and Hb_aliquot_ is the average Hb concentration of the RBC aliquots at a Hct of 40%. Hemolysis and cellular fragility tests were also performed after NAC supplementation (2.5 mM) or incubation for 24 h at 37 °C.

### 2.4 Analysis of microparticles

Enumeration and characterization of supernatant and circulating microparticles was performed by flow cytometry in aliquots of supernatant or plasma produced after two “light” spins of citrated blood at 20 °C. Microparticles were identified by size (<1 μm), RBC origin (FITC-conjugated anti-CD235) and PS exposure (AnnexinV-positive, AnnV^+^) as previously described [8].

Microparticles-associated procoagulant activity was estimated through thrombin generation measurement by using a functional Elisa assay kit (Zymuphen MP-activity) according to the manufacturers’ instructions (Hyphen BioMed, Neuville-sur-Oise, France).

Microparticles isolation by high speed centrifugation of the supernatants (37,000×*g* for 1 h) of G6PD^−^ (*n*=3) and control (G6PD^+^) units (*n*=2) on day 42 of storage and the subsequent measurement of carbonylated proteins in the vesicular pellet (Oxyblot detection kit, Millipore, Temecula, CA) were performed as previously described [2], [9]. The relative membrane expression of each component was estimated by scanning densitometry. HRP-conjugated antibodies to rabbit and to mouse IgGs were from GE Healthcare (Buckinghamshire, UK) and DakoCytomation (Glostrup, Denmark), respectively. Western lighting Plus ECL was from Perkin Elmer (CA, USA).

### 2.5 Malate estimation by metabolomics analysis

Metabolomics analyses were performed as previously reported [10], [11] and extensively described in (“Glucose 6-phosphate dehydrogenase deficient subjects may be better “storers” than donors of red blood cells”) [1].

### 2.6 NAC supplememtation

NAC (Sigma-Aldrich, Munich, Germany) was used in the majority of experiments as an exogenous antioxidant factor. Briefly, 2.5 mM NAC was added in RBC aliquots at the middle of storage period (day 21) and its effect was measured at the last day of storage (day 42).
